# Energy homeostasis in leptin deficient *Lep^ob/ob^* mice

**DOI:** 10.1371/journal.pone.0189784

**Published:** 2017-12-20

**Authors:** Alicja A. Skowronski, Yann Ravussin, Rudolph L. Leibel, Charles A. LeDuc

**Affiliations:** 1 Institute of Human Nutrition, Columbia University, New York City, New York, United States of America; 2 Department of Medicine, Columbia University, New York City, New York, United States of America; 3 Department of Pediatrics, Columbia University, New York City, New York, United States of America; 4 Obesity Research Core, Columbia University, New York City, New York, United States of America; Hospital Infantil Universitario Nino Jesus, SPAIN

## Abstract

Maintenance of reduced body weight is associated both with reduced energy expenditure per unit metabolic mass and increased hunger in mice and humans. Lowered circulating leptin concentration, due to decreased fat mass, provides a primary signal for this response. However, leptin deficient (*Lep*^*ob/ob*^) mice (and leptin receptor deficient Zucker rats) reduce energy expenditure following weight reduction by a necessarily non-leptin dependent mechanisms. To identify these mechanisms, *Lep*^*ob/ob*^ mice were fed ad libitum (AL group; n = 21) or restricted to 3 kilocalories of chow per day (CR group, n = 21). After losing 20% of initial weight (in approximately 2 weeks), the CR mice were stabilized at 80% of initial body weight for two weeks by titrated refeeding, and then released from food restriction. CR mice conserved energy (-17% below predicted based on body mass and composition during the day; -52% at night); and, when released to *ad libitum* feeding, CR mice regained fat and lean mass (to AL levels) within 5 weeks. CR mice did so while their *ad libitum* caloric intake was equal to that of the AL animals. While calorically restricted, the CR mice had a significantly lower respiratory exchange ratio (RER = 0.89) compared to AL (0.94); after release to *ad libitum* feeding, RER was significantly higher (1.03) than in the AL group (0.93), consistent with their anabolic state. These results confirm that, in congenitally leptin deficient animals, leptin is not required for compensatory reduction in energy expenditure accompanying weight loss, but suggest that the hyperphagia of the weight-reduced state is leptin-dependent.

## Introduction

In rodents and humans with an intact leptin axis, changes in body weight imposed by either overfeeding or dietary restriction are rapidly reversed when *ad libitum* feeding is resumed by coordinate reduced energy expenditure and hyperphagia [1, 2]. In addition, when non-obese individuals undergo liposuction, adipose tissue is redistributed to other depots, leading to the same overall level of adiposity within a year [3]. These observations support the concept that individuals regulate their body weight and adiposity at a level (“set point”) influenced by genetics, developmental factors, and the environment. In a weight-reduced state, both humans [4, 5] and mice [6, 7] become hyperphagic and their energy expenditure decreases more than predicted by their smaller body size; both phenotypes are largely reversed by physiological doses of exogenous leptin [8]. These observations suggest that reduction of circulating leptin is a major signal responsible for the metabolic and behavioral responses that lead to regain of lost weight. However, despite their congenital absence of leptin, *Lep*^*ob/ob*^ mice reduce their energy expenditure when calorically restricted [9]. Following surgical excision of fat, *ad libitum*-fed *Lep*^*ob/ob*^ mice regain lost fat in other depots and eventually achieve the same level of adiposity as sham-operated *Lep*^*ob/ob*^ mice [10].

Like *Lep*^*ob/ob*^ mice, the A-ZIP/F1 mice have greatly reduced circulating leptin and enter torpor when fasted [11]. But, unlike *Lep*^*ob/ob*^ mice, the leptin deficiency of A-ZIP/F1 mice is due to absence of white adipose tissue. Administration of leptin prevents fasting- induced torpor and hypothermia in *Lep*^*ob/ob*^ mice [12], but not in A-ZIP/F1 mice, suggesting that, in addition to low leptin, torpor in mice may be dependent on adipose signal(s) that are independent of leptin [11]. Doring *et al*. demonstrated that food restricted wild type mice reduce energy expenditure with circadian lows in metabolic rate at night and when supplemented with leptin, increase energy expenditure by increasing metabolic rate only at these circadian minima [13]. Bolze *et al*. administered modified long-acting leptin (PASylated leptin) to *Lep*^*ob/ob*^ mice and pair-fed another group of *Lep*^*ob/ob*^ mice. The leptin treated mice lost significantly more weight than the pair fed mice, indicating that in addition to energy intake leptin acted on energy expenditure; they estimated that the anorexigenic effect of leptin contributed to 75% of the weight loss and the energy expenditure accounted for 25% of the weight loss [14].

In the current study we found that *Lep*^*ob/ob*^ mice, weight reduced by transient caloric restriction, regained lost weight to the level of *ad libitum* fed controls; unlike wild type mice that overeat for a period of time after release from restriction, the *Lep*^*ob/ob*^ mice did this without any transient overeating compared to *ad libitum* controls. These observations suggest that *Lep*^*ob/ob*^ mice regulate adiposity via a leptin-independent pathway. The striking resistance to dietary weight loss reported in rare humans homozygous for inactivating leptin mutations is consistent with this inference [15].

## Methods

### Animals

48 *Lep*^*ob/ob*^ six week old male mice fed *ad libitum* on (Purina LabDiet 5058) chow diet were obtained from Jackson Laboratory (Bar Harbor, ME). Upon arrival, mice were group-housed (3 mice per cage) with *ad libitum* access to chow and water for a two week acclimation period. Throughout the study, animals were maintained at room ambient 22–24°C with a 12-h dark-light cycle (lights on at 0700h) in a pathogen-free barrier facility. The protocol was approved by the Columbia University Institutional Animal Care and Use Committee.

### Pilot experiment

6 *Lep*^*ob/ob*^ male mice at 8 weeks of age were either weight reduced by 20% (CR, n = 3) via caloric restriction, or fed chow *ad libitum* (AL, n = 3). Body weight and food intake were monitored daily. Upon reaching 80% of their initial body weight, CR mice were released to *ad libitum* feeding.

### Large cohort—study design

**Fig 1:**
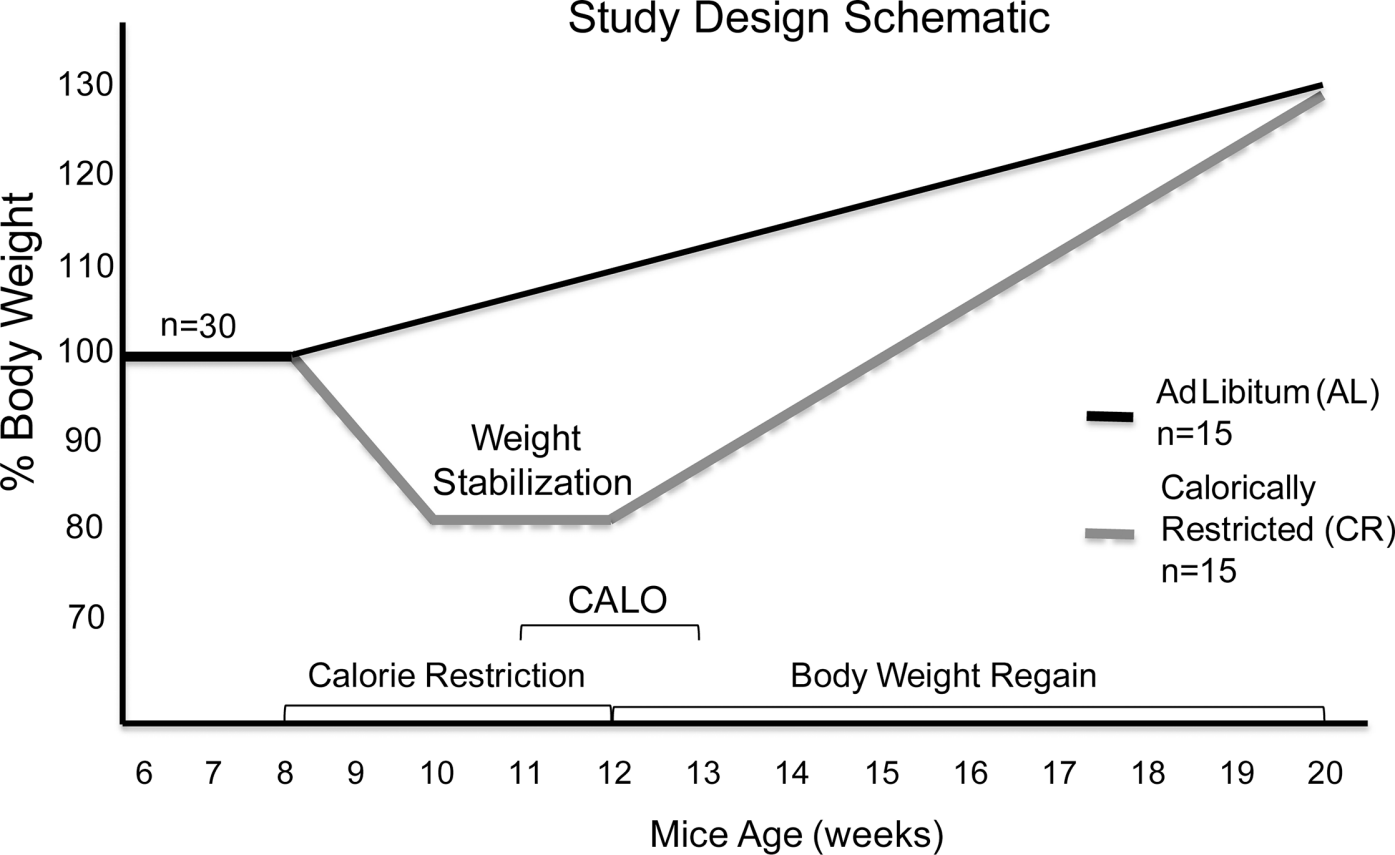
Study design schematic. Twenty percent weight reduction was achieved by feeding mice 1g of chow daily. During the weight maintenance phase, food intake was increased to 2-3g per day per mouse (the amount of food was adjusted daily when % of initial body weight of a mouse deviated from 80% by more than 2%). Calorically-restricted mice were provided with food twice daily, 1/3 of the total daily calories in the morning (09:00–9:30h) and 2/3 in the evening (18:00–18:30h). Body weight and food intake were monitored daily. During the second week of weight maintenance, mice were placed individually in metabolic cages to assess their energy expenditure (EE; TSE calorimetry system). They were then released to *ad libitum* feeding and EE was measured for another week. Mice were monitored for eight weeks until the body weight of the previously calorie restricted group reached that of with the never-restricted controls at which point mice were sacrificed.

Study design is outlined in **Fig 1**. After the two week acclimation period, at 8 weeks of age, half of the mice were assigned to the control group and fed *ad libitum* chow (AL group; n = 21); the other half were calorie restricted to achieve 80% of initial body weight (CR group, n = 21). Cages were assigned so that the two groups had no differences in starting weight, adiposity, or variance of those variables. After achieving 20% weight loss (approximately 2 weeks), the CR *Lep*^*ob/ob*^ mice were stabilized at 80% of initial body weight, by titrated feeding, for two weeks and then released from food restriction during the body weight re-gain phase.

### Body weight, body composition, food intake, and body temperature

BW was measured (± 0.1 g) daily in all mice throughout the experiment using an Ohaus Scout Pro 200g scale (Nänikon Switzerland, between 09:00–09:30h). Body composition [fat mass (FM), fat-free mass (FFM), and extracellular fluid] was measured by time-domain-NMR (Minispec Analyst AD; Bruker Optics, Silberstreifen, Germany) [16] once per week. Food was placed on the top of the cages and food intake was recorded daily for all mice throughout the study. Since mice were housed 3 per cage, food intake data was monitored on a per cage basis. Group housing the *Lep*^*ob/ob*^ mice (3 per cage) reduced stress and the individual mice lost body weight at the same rate. 24 hour core body temperature of mice was measured during the first week of the weight maintenance phase (**Fig 1**). Rectal core body temperature was measured every 3 h for 24 h using a Thermalert Monitoring Thermometer starting at 1500.

### Plasma assays

Blood was collected on ice using heparinized tubes (Fisherbrand). Plasma was isolated by centrifugation for 20 min at 2,000 x g at 4°C and frozen at −80°C until assay. Mice were bled at 11 weeks of age prior to transfer to the calorimeters (**Fig 1**). Blood from CR mice was collected before feeding (fasted overnight) while AL group was bled in a fed state. Insulin was assayed using Rat/Mouse ELISA kit (Mercodia) and glucose using Autokit Glucose (Wako).

### Energy expenditure

Energy expenditure was measured individually with a LabMaster-CaloSys-Calorimetry System (TSE Systems, Bad Homburg, Germany) from 1 week before the release of the CR group through 1 week of the body weight regain phase (**Fig 1**). Concentrations of cage oxygen (O_2_) and carbon dioxide (CO_2_) were measured from every mouse every 17 minutes during the two weeks of calorimetry. To mitigate the effects of stress associated with exposure of mice to a new environment, the first 24 hours of data were excluded. Resting energy expenditure (REE; kcal/24hr) was defined as the lowest one-hour period of energy expenditure during the day; this value was extrapolated to 24 hours. This value was taken instead of the lowest period in 24 hours because, for the CR mice, the 24 hour nadir occurred during nocturnal torpor [9] which depressed the REE. Torpor suppression of energy expenditure in CR mice was calculated by subtracting the lowest one-hour period of energy expenditure during the night from the REE and multiplying it by the percent of time the mouse spent in torpor during any given 24-hour period. Non-resting energy expenditure (NREE; kcal/24hr) was calculated by subtracting REE from TEE and adding torpor suppression (for mice that entered torpor) (NREE_CR_ = TEE–REE + Torpor suppression; NREE = TEE-REE when no torpor).

Physical activity was determined with an infrared beam system integrated with the TSE LabMaster system. Total activity (number of infrared beam breaks) in X, Y, and Z axis was recorded in 17-minute time intervals and a corresponding TEE was measured at the end of each interval. To assess the instantaneous TEE as a function of activity, every reading for every mouse was categorized as occurring during the day (between 7am and 7pm) or night (between 7pm and 7am) and then combined by group (AL, CR, or POST CR). To determine if energy expenditure was conserved at a given activity level in the groups, each group was sorted by day [0700–1900] and night [1900–0700] from lowest to highest activity [beam breaks] with contemporaneously measured TEE. Measured TEE and beam breaks were assigned to bins of 100 beam breaks. Means and standard deviations of both activity and TEE were determined for each bin.

Average weekly energy expenditure after release from calorie restriction until the body weight of CR mice recovered to AL levels was calculated for individual mice per mean value for each cage using the energy balance equation: TEE = FI − (Δ somatic Fat Energy + Δ somatic Fat−Free Energy) [17]. Weekly food intake and weekly change in fat and lean mass were used in the calculation.

### Statistical analysis

Data are expressed as means ± SEM. Statistical analysis was performed using GraphPad PRISM software. Student t-tests (2-tailed) were conducted to compare AL and CR groups. P alpha < 0.05 was taken as significant. To determine whether the increased energy expenditure efficiency in the CR and post-CR state occurred in the dark or light cycle and if they were due to differences in physical activity, plots of TEE as a function of movement were made. Regression of instantaneous TEE as a function of movement was analyzed by first smoothing the data with a Lowess curve (with 5 points per smoothing window) then calculating the difference in TEE between the groups followed by a repeated measure one-way ANOVA and post hoc Bonferroni's multiple comparisons test to determine the difference between the AL and CR (during and post CR) curves.

Several mice were excluded from the analysis. Two mice died during body composition measurement (one from each group). One mouse was found dead in its cage (CR group). Four mice (two from each group) were removed from the study because they were not gaining weight and dropped below the 3^rd^ standard deviation for weight of the group.

## Results

### Pilot experiment

**Fig 2:**
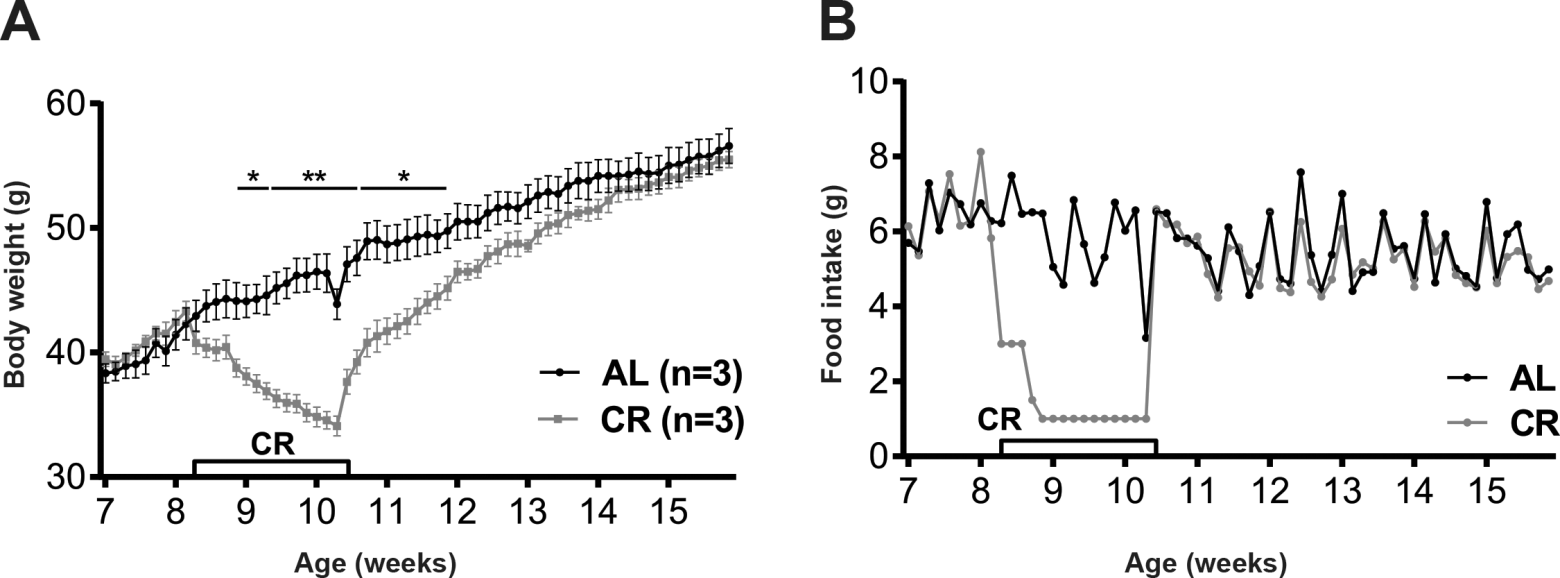
Body weight and food intake of AL and CR mice in a pilot study. (A) Mean body weight ±SEM (g) and (B) Mean 24h food intake in mice fed *ad libitum* throughout the study (AL) and mice calorically restricted to 80% of initial body weight then released to *ad libitum* feeding. P values: *<0.05, **<0.01.

At baseline (7 week of age) *Lep*^*ob/ob*^ mice were similar in body weight (**Fig 2A**) and consumed the same number of calories per day (**Fig 2B**). One cage of three mice was restricted to 1 g of food per day per mouse and lost weight at an average rate of 0.6 g per day, these CR mice achieved 20% weight loss in 2 weeks. In parallel, AL mice gained body weight at a steady rate of approximately 0.3 g per day, for a total weight gain of 4 g over 2 weeks. CR and AL groups had significantly different body weights throughout the calorie restriction phase (**Fig 2A**). After release from caloric restriction, CR mice immediately returned to their pre-CR food intake and never overate relative to the never-restricted control mice (**Fig 2B**). The CR group re-gained their lost body weight within 8 days of *ad libitum* feeding (**Fig 2A**).

Despite the re-gain of body weight, the CR group had a significantly lower BW compared to AL group since the *ad libitum* mice continued to increase body weight throughout the experiment (**Fig 2A**). Mice were allowed to continue eating *ad libitum* for another 4 weeks. By the end of the study, CR mice fully caught up with AL group and did so without any detectable difference in food intake. Neither energy expenditure nor body temperature were monitored in this pilot study but body weight and food intake data suggest that weight reduced mice had decreased energy expenditure.

### Large cohort experiment

#### Body weight and composition

**Fig 3:**
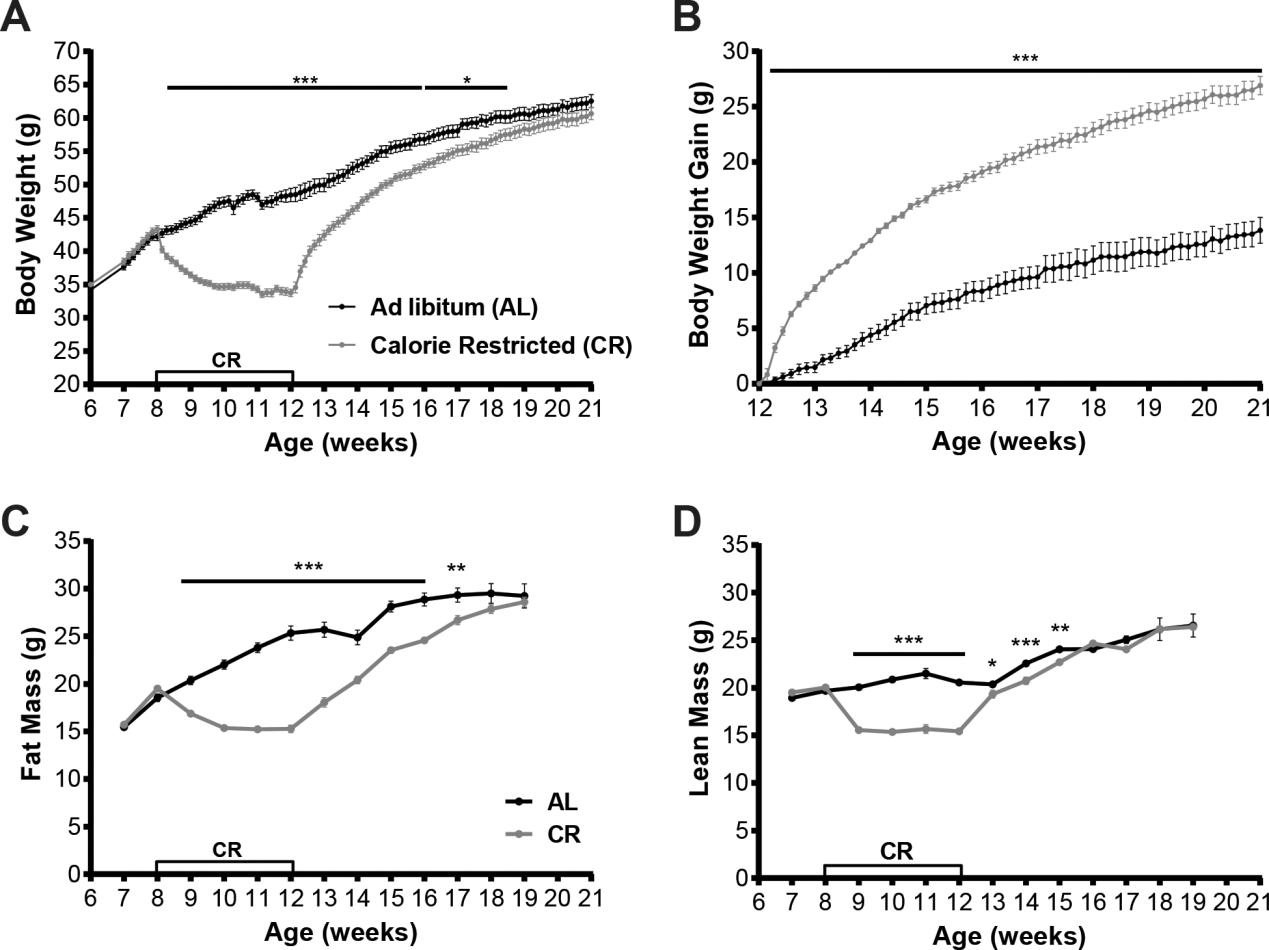
Body weight and composition in AL and CR mice. (A) Mean body weight, (B) body weight gain, (C) fat mass, (D) lean mass ±SEM (g) in mice fed *ad libitum* throughout the study (AL) and mice calorically restricted (CR) to 80% of initial body weight then released to *ad libitum* feeding. P values: *<0.05, **<0.01, ***<0.001.

**Fig 4:**
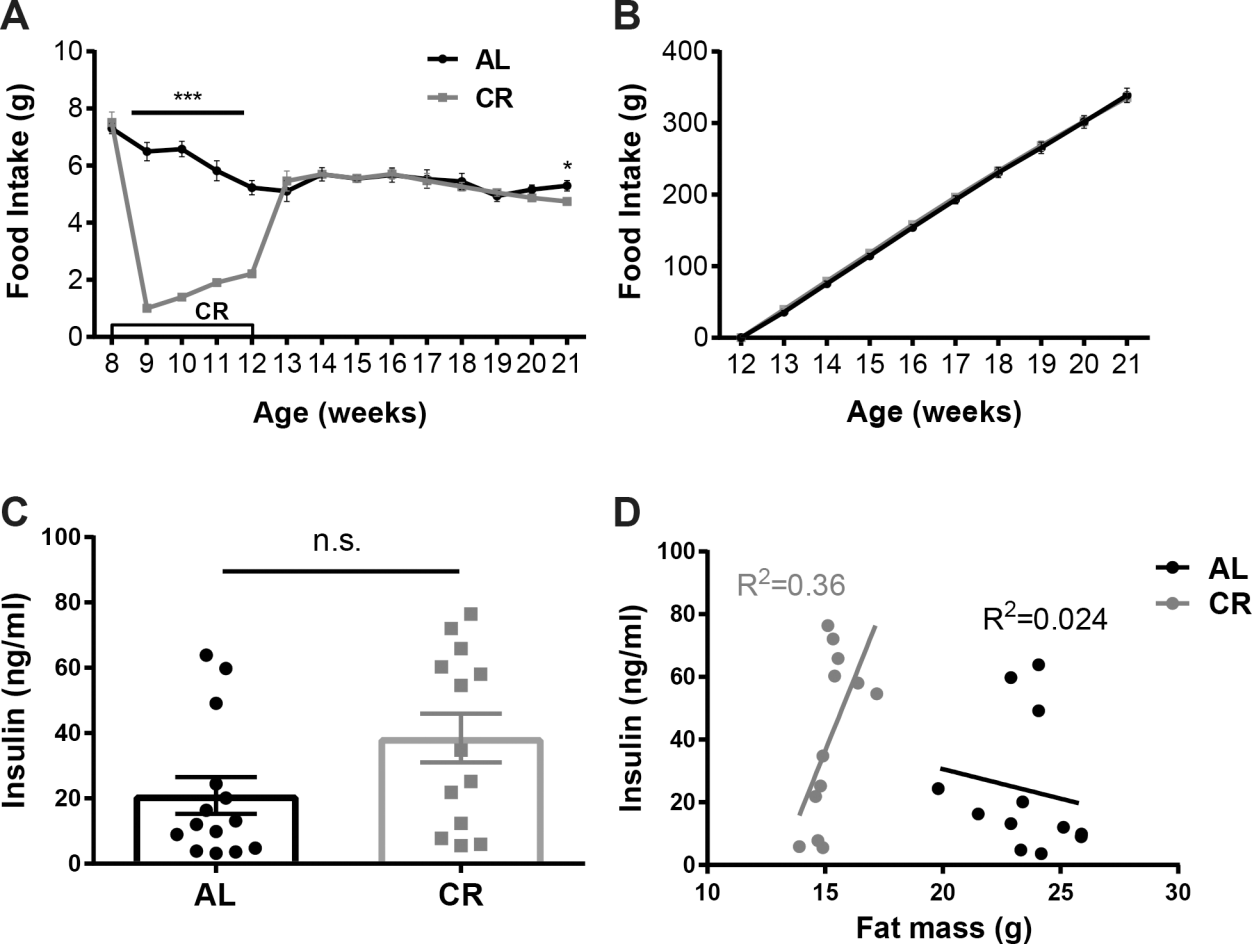
Food intake, plasma glucose and insulin in CR and AL mice. (A) Mean 24h food intake ±SEM (g) and (B) Cumulative food intake over 8 weeks of body weight re-gain in mice fed *ad libitum* chow throughout the study (AL) and mice calorically restricted to 80% of initial body weight then released to *ad libitum* feeding. (C) Mean glucose and (D) insulin ±SEM in *ad libitum* fed (AL) or calorically restricted (CR) mice measured at 12 weeks of age while CR mice were calorically restricted to maintain 80% of initial body weight. (E) Regression of circulating insulin concentrations against fat mass in the AL and CR groups of mice at 11 weeks of age while CR were weight stable at the reduced body weight. P values: ***<0.001.

Upon arrival from JAX at 6 weeks of age the average weight of the *Lep*^*ob/ob*^ male mice was 34.72±0.38 g. At 8 weeks of age, *Lep*^*ob/ob*^ mice were assigned to either a calorie restriction group (CR, n = 15) or *ad libitum* fed group (AL, n = 15) so that the groups did not differ in mean body weight (AL: 42.19±0.6 g; CR: 43.30±0.54 g; p = 0.19; **Table 1**, **Fig 3A**) or body composition (fat mass, AL: 18.54±0.40 g; CR: 19.5±0.30 g, p = 0.07; and lean mass, AL: 19.69±0.27 g; CR: 20.03±0.22 g, p = 0.35; **Table 1, Fig 3C and 3D**). The mean baseline 24h chow intake (3.45 kcal per g) per mouse during the week prior to the start of CR was 7.30±0.18 g and 7.51±0.36 g in AL and CR groups, respectively (**Table 1, Fig 4A**). After the first day of CR, body weight was significantly lower in the CR mice compared to AL mice (AL: 42.70±0.62 g; CR: 40.23±0.47 g; p = 0.0047; **Fig 3A**) and remained significantly lower throughout the calorie restriction phase. Upon reaching 20% weight loss, body weight of CR group was 34.75±0.43 g compared to 46.73±0.80 g in AL group (**Table 1,** p <0.001). Both fat mass and lean mass were significantly decreased in CR mice compared to AL (fat mass, AL: 22.03±0.48 g; CR: 15.37±0.23 g, p<0.001; lean mass, AL: 20.87±0.24 g; CR: 15.37±0.32 g, **Table 1,** p<0.001). At 10 weeks of age, CR mice entered the body weight stabilization phase to maintain reduced body weight for two weeks. Food intake in the CR group during this phase was titrated up from 1 g per day per mouse to 1.5–2.5 g per day. Average 24h food intake of AL group mice at 10 weeks of age was 6.58±0.27 g (**Table 1, Fig 4A**).

**Table 1:** Body weight, body composition, and food intake of AL and CR mice.

	Baseline (8 wks old)			During CR (10 wks old)			BW regained (18–18.5 wks old)		
	AL	CR	Pval	AL	CR	Pval	AL	CR	Pval
Body wt (g)	42.19±0.60	43.30±0.54	0.19	46.73±0.80	34.75±0.43	*<0*.*001*	60.18±0.95	57.55±0.91	0.06
Fat mass (g)	18.54±0.40	19.50±0.30	0.07	22.03±0.48	15.37±0.23	*<0*.*001*	29.52±1.04	27.87±0.47	0.12
Lean mass (g)	19.69±0.27	20.03±0.22	0.35	20.87±0.24	15.37±0.32	*<0*.*001*	26.13±1.19	26.16±0.32	0.98
Food intake (g)	7.30±0.18	7.51±0.36	0.61	6.58±0.27	1.40±0.08	*<0*.*001*	5.45±0.29	5.26±0.15	0.61

Mean body weight, fat mass, lean mass and food intake with SEM at baseline (8 weeks of age), during calorie restriction (10 weeks of age) and after CR group re-gained body weight (18.5 weeks of age; fat mass, lean mass and food intake measured at 18 weeks of age) in mice fed *ad libitum* throughout the study (AL) and mice calorically restricted to 80% of initial body weight then released to *ad libitum* feeding (CR).

At 12 weeks of age CR mice were released from calorie restriction and allowed to eat *ad libitum*. Mice in the CR group re-gained their lost body weight within 8 days of *ad libitum* feeding (CR: 43.19±0.71 g; **Fig 3A**). Despite the re-gain of their body weight, CR group had a significantly lower BW compared to AL group since the AL mice continued increasing body weight throughout time the CR mice were calorie restricted (AL: 50.60±1.04 g; p<0.001; **Fig 3A**). It took CR mice another 5 weeks of *ad libitum* feeding to achieve body weights not significantly different from AL mice (age 18 weeks; AL: 60.18±0.95 g; CR: 57.55±0.91 g; p = 0.058; **Table 1, Fig 3A**). Lean mass in CR group was regained sooner than body weight and fat mass. One week after release from caloric restriction, CR group mice regained most of their lean mass (AL: 20.36±0.26 g; CR: 19.33±0.41 g, p = 0.04) but were still significantly lower in lean mass than AL group. At 16 weeks of age the difference in lean mass between CR and AL groups was no longer significant (lean mass, AL: 24.07±0.20 g; CR: 24.66±0.27 g; p = 0.087; **Fig 3D**). Fat mass in CR group did not differ from AL group 6 weeks after release from calorie restriction (age 18 weeks; fat mass, AL: 29.52±1.04 g; CR: 27.87±0.47 g; p = 0.12; **Table 1, Fig 3C**).

Mice were sacrificed at 21 weeks of age, reaching final weights of 62.54±0.97 g and 60.64±0.92 g in AL and CR groups (p = 0.17), respectively.

#### Food intake

The mean baseline 24h food intake per mouse at 8 weeks of age (prior to start of CR) was 7.30±0.18 g and 7.51±0.36 g in AL and CR groups, respectively. During caloric restriction phase mice in CR group were provided with an average of 1 g of chow per day until at target weight. The mean 24-hour food intake during the first week after release from caloric restriction was 5.10±0.36 g and 5.46±0.35 g in AL and CR groups, respectively (p = 0.50; **Fig 4A**). Food intake was not different between AL and CR groups until the end of the study (**Fig 4A and 4B**). Over the 9-week period following the release of CR mice to *ad libitum* feeding (until the end of the study) cumulative food intake calculated for each group was 338.75±9.99 g and 335.86±7.40 g in AL and CR groups, respectively (p = 0.83; **Fig 4B**). Despite the difference in weight gained over this 9-week period (14.10 g in AL vs 26.92 g in POST CR), formerly calorie restricted mice did not increase their food intake relative to the control animals, suggesting that the CR animals re-gained weight solely as a result of their reduced energy expenditure in part due to their smaller size. Interestingly, for both groups, the amount of food ingested was inversely correlated to their age and weight. The mean 24h food intake at the beginning of the study, when mice were 8 weeks of age, was 7.41±0.20 g compared to 5.05±0.14 g by the end of the study at 21 weeks of age (p < .001; **Fig 4B**) despite weighing almost 50% more (~62 g vs ~43 g).

#### Plasma glucose and insulin concentrations

Plasma was obtained from AL and CR mice at 11 weeks of age, while CR group was weight stable at 80% of initial body weight. Despite a 14g lower body weight, plasma insulin in CR mice did not differ from the AL group (AL: 20.95±5.62 ng/ml; CR: 38.53±7.46 ng/ml; p = 0.07, **Fig 4C**). Plasma glucose was significantly lower in CR compared to AL group (AL: 332.6±39.21 mg/dl; CR: 149.5±26.50 mg/dl; p <0.001)–consistent with their feeding status (**Fig 4D**). Unlike mice with intact leptin axis [18, 19], leptin-deficient mice showed no correlation between fat mass and plasma insulin concentration in both the reduced weight and *ad libitum* fed states (**Fig 4E**).

**Fig 5:**
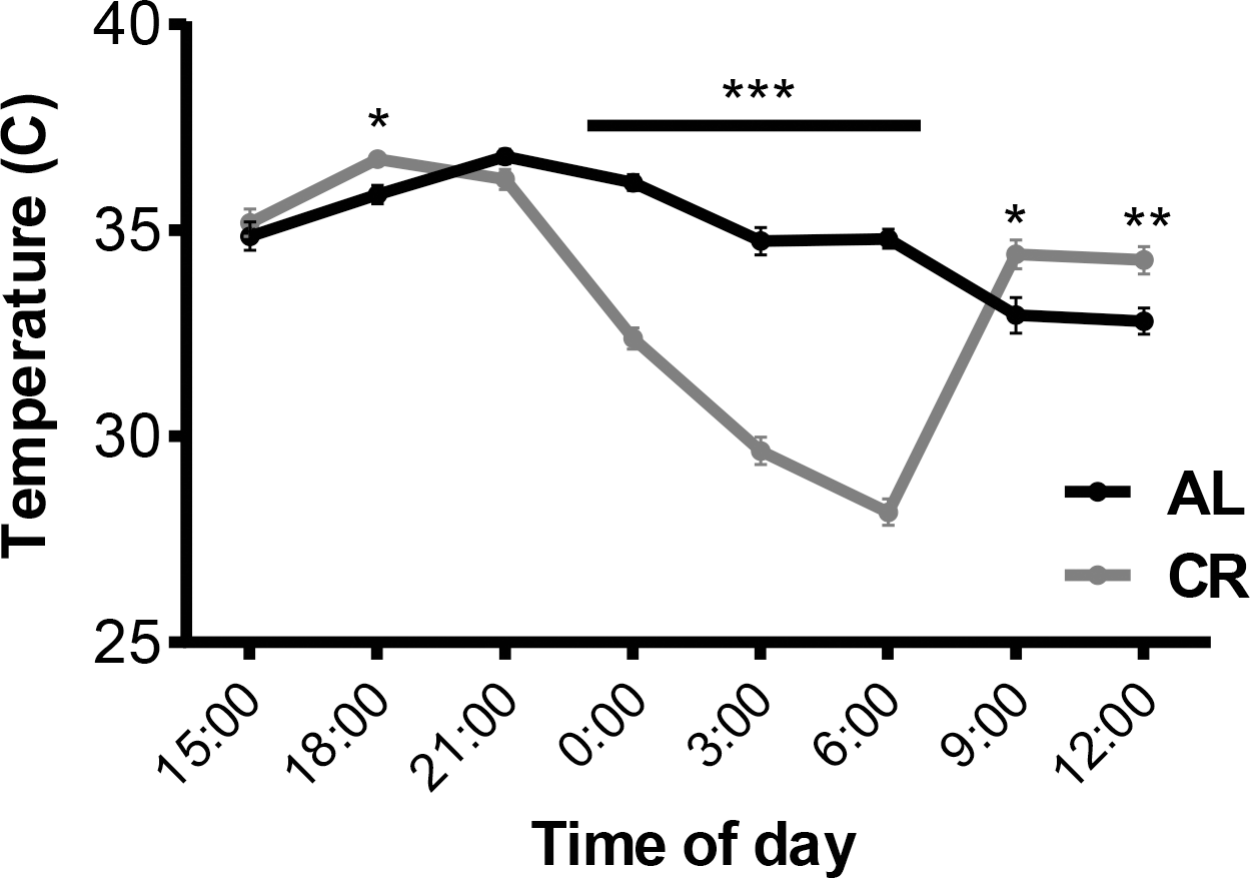
Body temperature of AL and CR mice. Body temperature of mice fed *ad libitum* chow throughout the study (AL) and mice calorically restricted to 80% of initial body weight (CR) measured during the weight maintenance segment of the CR phase.

*Body temperature*: AL mice maintained their body temperature between 32.8°C and 36.8°C with the lowest temperatures measured between 9am and 12pm. CR group maintained their body temperature within normal range during the day between 9 am and 9 pm. Between 9 pm and 6 am CR mice dropped their body temperature significantly; the lowest temperature recorded was 28.2°C at 6 am a few hours before the morning feeding (compared to 34.8°C in AL group, p<0.001; **Fig 5**).

**Fig 6:**
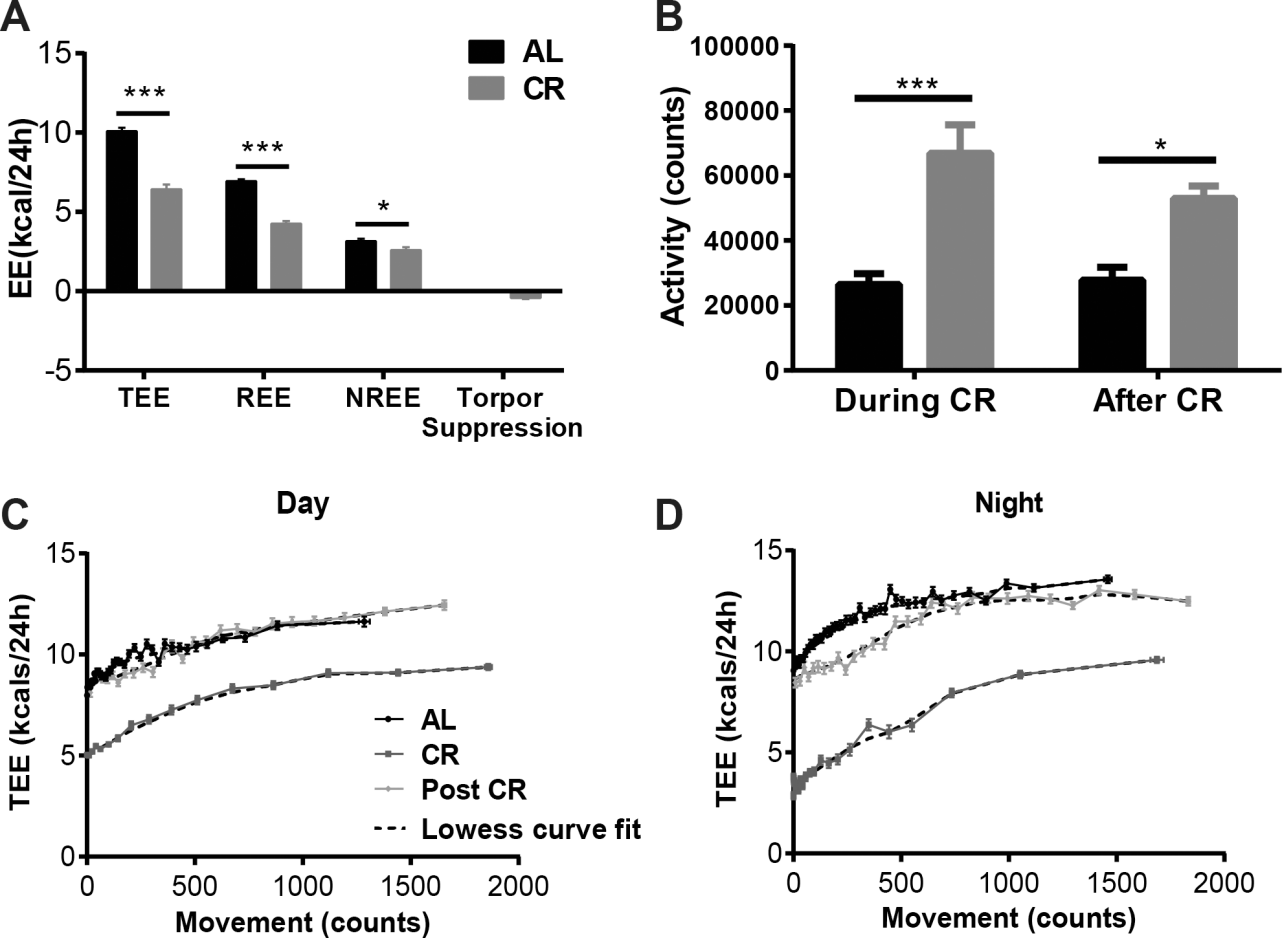
Energy expenditure and activity of AL and CR mice. (A) Energy expenditure during calorie restriction in mice fed *ad libitum* chow throughout the study (AL) and mice calorically restricted to 80% of initial body weight (CR). Energy expenditure during calorie restriction was measured in the TSE metabolic chambers. Included are the following: TEE–total energy expenditure, REE–resting energy expenditure, NREE–non resting energy expenditure and torpor suppression. (B) Physical activity in AL and CR mice during CR and after release to *ad libitum* feeding. Activity was measured in the TSE system. Regression of instantaneous TEE as a function of movement (C) during the day and (D) at night in mice fed *ad libitum* chow throughout the study (AL), mice calorically restricted to 80% of initial body weight (CR) and the CR group after release to *ad libitum* feeding. P values: *<0.05, ***<0.001.

*Energy expenditure*: During the reduced weight maintenance phase, absolute mean 24h total energy expenditure (TEE) was 36% lower in CR group compared to AL (AL: 10.10±0.20 kcal/24h; CR: 6.42±0.31 kcal/24h; p < 0.001; **Fig 6A**). Decreased TEE was accounted for by nocturnal periods of torpor in addition to decreased resting and non-resting energy expenditure (**Fig 6A**). In wild type mice, we have defined resting energy expenditure (REE) as the lowest one hour period of energy expenditure during each 24h period [6]. However, periods of torpor should not be included in estimates of REE. To address this problem, in calorie-restricted *Lep*^*ob/ob*^ mice, we defined REE as the lowest one-hour period of energy expenditure during the day (from calorimetry data, mice did not enter torpor during daytime). Absolute resting energy expenditure (REE) during reduced weight maintenance phase was decreased by 39% in CR compared to AL group (AL: 6.94±0.12 kcal/24h; CR: 4.26±0.17 kcal/24h; p < 0.001; **Fig 6A**); NREE was 18% lower in CR mice than in AL (AL: 3.16±0.15 kcal/24h; CR: 2.59±0.17 kcal/24h; p < 0.05; **Fig 6A**); additionally, CR mice conserved 0.43 kcal/24h by entering torpor. NREE was decreased in CR mice despite significantly higher total activity compared to AL mice (AL: 26,433±3,358 beam breaks per 24 hours; CR: 66,844±8,754 beam breaks per 24 hours; p < 0.001; **Fig 6B**). The regression of instantaneous TEE as a function of movement (individual mouse total activity during the period that was used for the TEE calculation) shows that this movement was achieved at lower energy cost (*i*.*e*. mice were more energetically efficient) during calorie restriction than in the AL group; this was true during both day and night (**Fig 6C and 6D**). CR mice were more active than AL mice during the day but were comparably active at night. The decreased body temperature and total activity at night suggests that CR mice entered torpor at night but had increased activity during the day, likely due to increased food seeking behavior.

**Fig 7:**
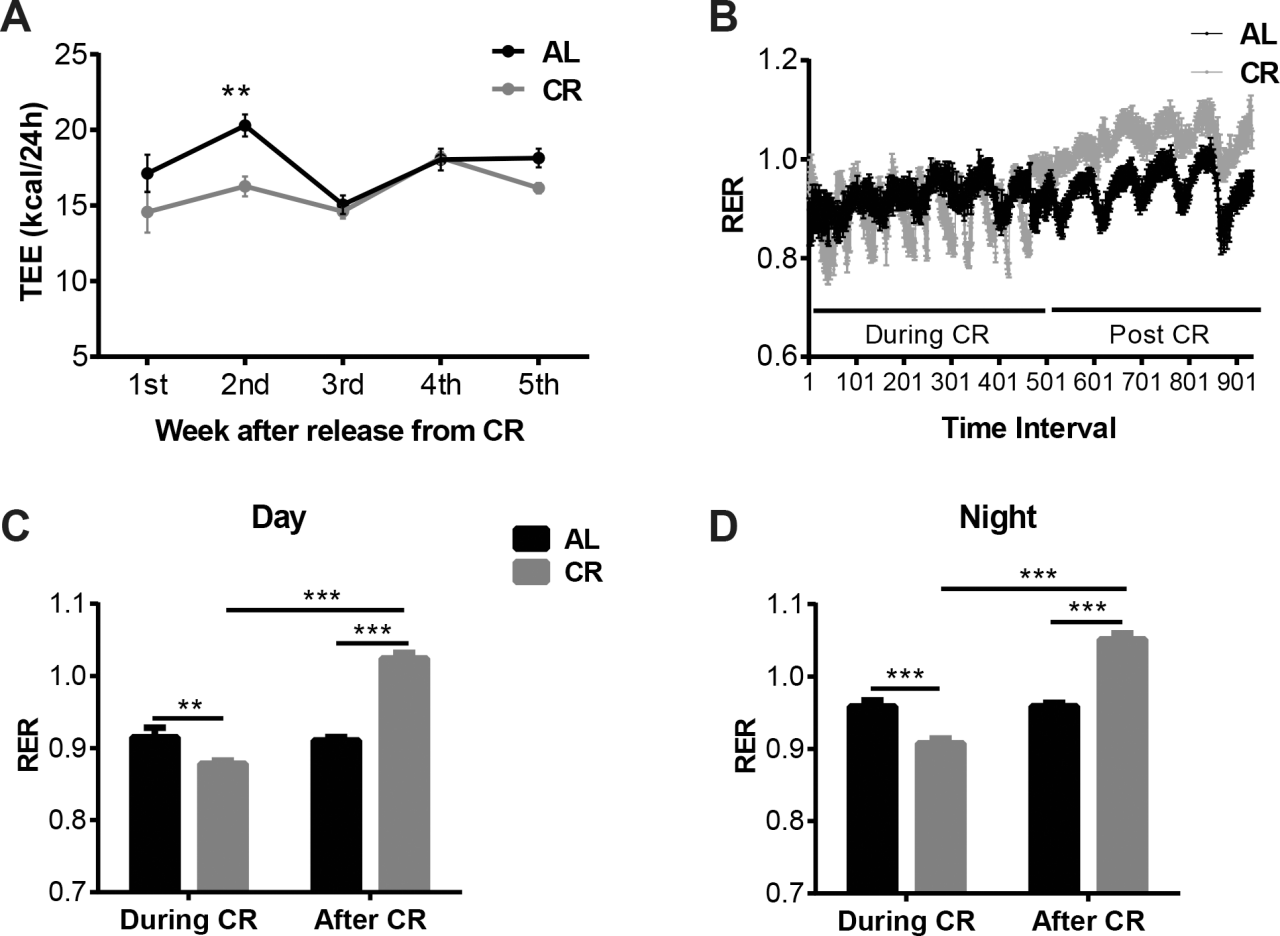
Total energy expenditure and respiratory exchange ratio. (A) Total energy expenditure after release from calorie restriction in mice fed *ad libitum* chow throughout the study (AL) and calorically restricted (CR) mice. TEE post-restriction was calculated using the energy balance equation: TEE = FI − (Δ somatic Fat Energy + Δ somatic Fat−Free Energy). (B) average respiratory exchange ratio (RER) measured at each time interval and (C) average 24-hour RER during the day and (D) and at night during and post calorie restriction in mice fed *ad libitum* chow throughout the study (AL) and mice calorically restricted then released to *ad libitum* feeding. P values: **<0.01, ***<0.001.

Energy expenditure was measured for 6 days after the CR group was released to *ad libitum* feeding and entered the body weight re-gain phase. The TSE indirect calorimetry system uses the Weir’s equation to calculate the heat production from the volume of O_2_ consumed and CO_2_ produced which assumes that the respiratory exchange ratio (RER) of animals is between 0.7 and 1.0 [20]. During the body weight re-gain phase, the CR mice were actively gaining muscle and fat; their RER at that time was above 1, consistent with their anabolic state (1.03 on average but 1.05 at night). As a result of RER being outside of range for the Weir equations heat estimate, the TEE obtained from the TSE system is inaccurate. This data is included in S1 Fig. Therefore, we calculated the mean total energy expenditure for individual mice using energy balance equation [TEE = FI − (Δ somatic Fat Energy + Δ somatic Fat−Free Energy)] [17] in AL and CR mice in the 5 weeks following release from caloric restriction until the CR group regained lost weight (**Fig 7A**). Using this method, TEE in CR mice was significantly lower than in AL group during the 2^nd^ week post release from CR (**Fig 7A**). Results were similar when average energy expenditure was calculated on a per cage basis (average of 3 mice per cage).

In addition, the regression of TEE as a function of movement (total activity) shows that, after CR mice were released to *ad libitum* feeding, they were still significantly more efficient with regard to the energy cost of motion than AL mice but this increased efficiency was apparent only during the dark phase (**Fig 6C and 6D**). The total activity increase found in the CR compared to the AL mice persisted despite *ad libitum* access to food (AL: 327.2±47.0 beam breaks; CR: 622.6±46.0 beam breaks; p < 0.001; **Fig 6C**).

Respiratory exchange ratio (RER or RQ) fluctuates throughout the day but the amplitude was more pronounced in the CR state compared to AL (**Fig 7B**). The 24 hour RER of the weight stable CR mice was equal to the diet quotient of the chow (both 0.89). At night, when AL mice primarily eat, the RER was elevated and it fell significantly during the day when mice eat less. In the CR state, mice ingest their food rapidly after the twice-daily feeding. This pattern of feeding imposes on these animals extended periods of fasting, leading to reduced RER both during the day and the night compared to the AL mice (**Fig 7B–7D**). RER increased dramatically when the CR mice were released from caloric restriction; the POST CR mice had consistently higher RER at every time point (**Fig 7B–7D**).

**Fig 8:**
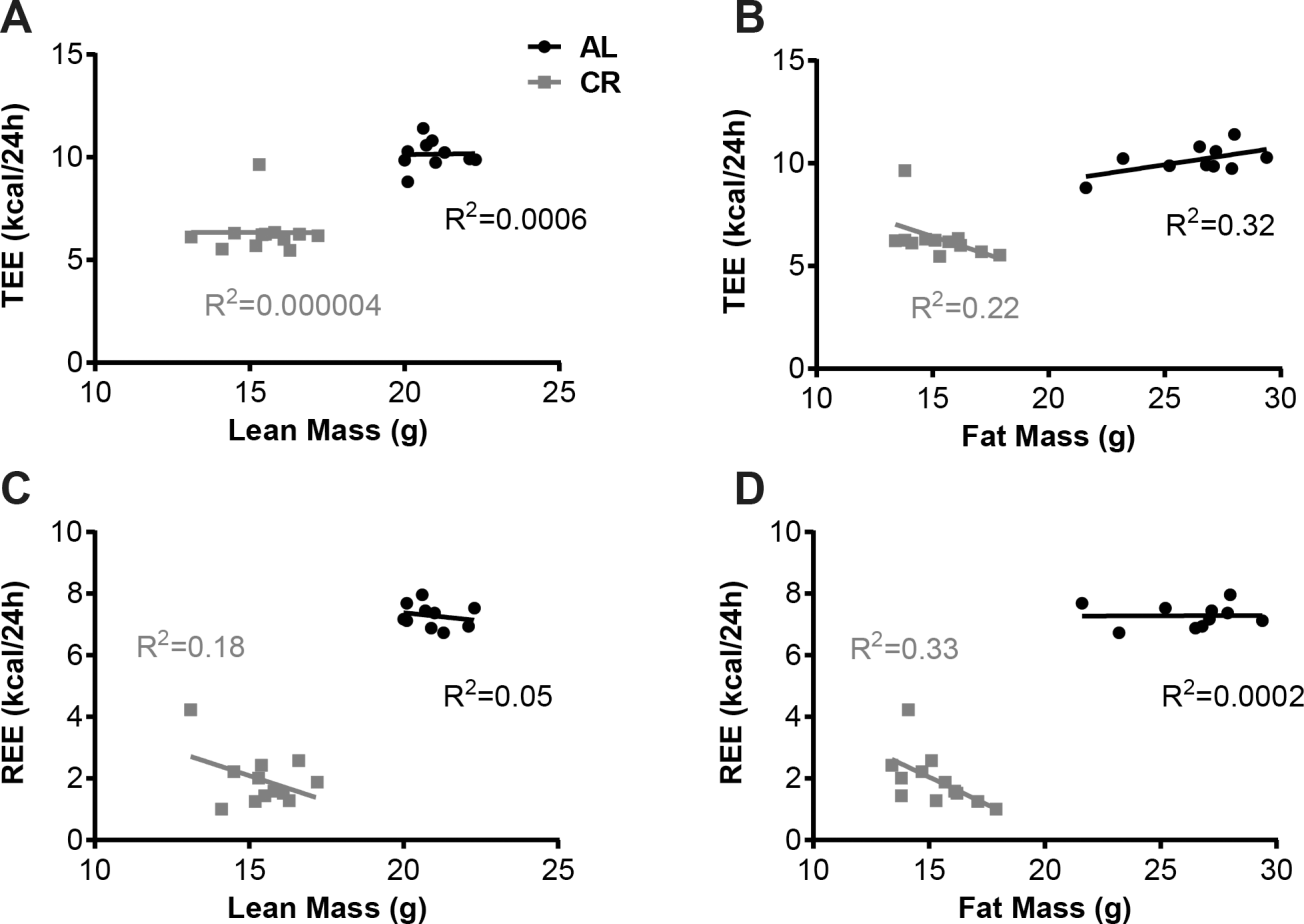
Correlations of energy expenditure with body composition in AL and CR mice. Regression of (A, C) lean mass and (B, D) fat mass against (A, B) total and (C, D) resting energy expenditure in the AL and CR groups of mice during the weight maintenance segment of the CR phase.

In the *Lep*^*ob/ob*^ mice at reduced weight and during *ad libitum* re-feeding there was no correlation between total or resting energy expenditure and lean mass, fat mass, or both combined over the ranges observed (**Fig 8)**. In humans and rodents with an intact leptin axis, energy expenditure is highly correlated with metabolic mass [6].

## Discussion

In humans and rodents with intact leptin signaling, weight reduction imposed by caloric restriction reduces energy expenditure more than predicted by remaining metabolic mass [4, 6, 8, 9, 21]. We find that this response is significantly amplified in leptin-deficient mice. The TEE of weight-reduced *Lep*^*ob/ob*^ mice in this study was almost 40% lower than the *ad libitum* fed mice. These effects were conveyed by reductions in REE (approximately 73% of the reduction), enhanced efficiency of motion (about 16%), and periods of torpor (about 12%). About 50% of energy expenditure of a mouse maintained at room temperature of 22–24°C is used to maintain body temperature [9]. Under conditions of restricted access to food, *Lep*^*ob/ob*^ mice dramatically lower their body temperature at night and enter torpor; energy expenditure in CR mice was 39% less than in *ad libitum* fed controls. In comparison, diet induced obese wild type mice reduce resting energy expenditure by 25% when calorie restricted [9]. In wild type mice, circulating leptin follows a circadian rhythm that peaks in the middle of the night [22] and this peak coincides with the trough in body temperature that was detected in the CR *Lep*^*ob/ob*^ mouse.

In *Lep*^*ob/ob*^ mice, REE was calculated as the lowest energy expenditure during the day (*Lep*^*ob/ob*^ mice did not enter torpor with the lights on) and torpor suppression is the amount of energy that is conserved at night beyond the REE due to torpor-related lowering of body temperature. Others have reported that *Lep*^*ob/ob*^ mice enter torpor at night which extends into the light phase; however, in these studies mice were fed only once a day [14] or were fasted overnight prior to energy expenditure measurement [11]. In the current study, mice were fed 1/3 of the daily ration in the morning and 2/3 in the evening; the mice only entered torpor during the dark cycle. Defining NREE as TEE minus REE plus torpor suppression, the restricted *Lep*^*ob/ob*^ mice have approximately the same NREE as the AL mice, despite 2.5 times greater movement (beam breaks). The increased physical activity persists in the CR mice after they are released to *ad libitum* feeding; we speculate that this persistence is due to increased food seeking behavior.

In AL mice, physical activity is highest at night and reduced during the day. CR mice display an inverted pattern in which highest activity is during the day while at night they enter torpor and show almost no physical activity. As reported here, caloric restriction resulted in a significant 2.5-fold increase in physical activity in *Lep*^*ob/ob*^ animals. An increase in movement is also apparent in food restricted wild type mice but the magnitude of the effect is much smaller (~20%) [6].

Data in Fig 6C and 6D demonstrate that the CR mice, prior to release, are more energy efficient in spontaneous physical activity. Once released from caloric restriction, this increased efficiency is restricted to the lights-off period despite these mice no longer going into torpor (as seen by calorimetry).

The *ad libitum* fed formerly CR *Lep*^*ob/ob*^ mice gained weight at a faster rate than the AL mice despite no detectable difference in energy intake. The TEE in the formerly restricted mice (estimated using changes in somatic energy content over a 7 day period [23]) was significantly lower in CR animals during the second week of *ad libitum* feeding, but not thereafter.

Wild type mice are hyperphagic following release from caloric restriction [7, 24]. On the first day post restriction–while wild type calorie restricted mice are significantly lighter (-25% body weight) than *ad libitum* fed controls–food intake is almost doubled [7]. This relative hyperphagia decreases over the ensuing 7 days, at which point the intake of the weight-reduced animals equals that of the controls [7]. In *Lep*^*ob/ob*^ mice reported here, food intake in the immediate post restriction period was identical in the released mice and the never-restricted AL groups despite the increased food seeking behavior of the formerly restricted mice. Fig 4 shows that total food intake does not increase as L*ep*^*ob/ob*^ mice age and gain weight. Additionally, Fig 8 demonstrates that there is no correlation between somatic mass and energy expenditure in either the CR or the AL state in Lep^*ob/ob*^ animals over the range of this study. Since there is no relationship between mass and energy expenditure in Lep^*ob/ob*^ animals, CR mice, released to *ad libitum* feeding, should have gained weight at the same rate as the never-restricted mice unless there is an increase in energy efficiency that persists after release. The absence of post-restriction hyperphagia is not due to mice reaching their physical (e.g. stomach capacity) maximum daily energy intake since the same mice ate greater amounts earlier in the study (7.51 ± 0.36 g per day at 8 weeks of age vs. 5.46 ± 0.35 g per day at 13 weeks of age after release from restriction; p<0.01). Another argument against *Lep*^*ob/ob*^ mice having reached a physical maximum of food intake is that *Lep*^*ob/ob*^ mice are capable of further increasing food intake when suitably provoked. For example, the FAT-ATTAC transgenic mouse segregates for a myristoylated caspase 8-FKBP fusion protein enabling adipocyte apoptosis to be induced by administration of a chemical dimerizer for 1–2 weeks [25]. *Lep*^*ob/ob*^ mice segregating for this transgene, following administration of the dimerizer, increase daily food intake of chow from 5 g to 9 g (6 weeks of treatment) [25].

As extensively discussed in literature, expressing food intake in terms of calories per gram of body weight is problematic especially when the animals being compared differ significantly in body mass. For example, normalizing food intake of a *Lep*^*ob/ob*^ mouse to body weight would suggest that such mouse is hypophagic relative to a wild type mouse [26]. Others have recommended that energy intake be analyzed by multivariate regression [27, 28]; or that both energy intake and expenditure be normalized using the same method [29, 30]. In the AL *Lep*^*ob/ob*^ group, food intake at 8, 9, and 10 weeks of age was significantly higher than food intake at the end of the study when mice were 21 weeks old and 20 grams heavier. This observation further supports the inference that food intake should not be normalized to body weight in these mice over the body weight ranges in this study. Energy intake and expenditure in the AL group decreased gradually from 8 to 12 weeks of age and appeared to stabilize after week 12; higher energy expenditure in *Lep*^*ob/ob*^ mice during this time may be related to maximal growth rate during this time. Despite the mice having 35% more mass at 21 weeks, there was no increase in calorie intake after 12 weeks of age; there was no correlation between energy expenditure and fat, lean, or total mass in any of the groups of leptin deficient mice over the range measured. In aggregate, these data suggest that leptin is required for the regulation of energy intake, but is not essential for regulation of energy expenditure.

*Lep*^*ob/ob*^ mice develop without exposure to leptin leading to congenital neuronal alterations [31, 32]. Compensatory pathways could develop as a result of congenital leptin deficiency and this leads to an important caveat that the responses to the weight perturbations reported here may be unique to these animals. In addition to this shortcoming, there could be a difference in the extraction efficiency in the feces during and after caloric restriction. Unfortunately, feces was not collected for bomb calorimetry. The current study is limited in the ability to look at genetic factors (aside from leptin) that can alter the response to caloric restriction since all the mice studied were the same strain. In rats there is a divergent response to refeeding after caloric restriction between Sprague Dawley and Long Evans rats. Sprague Dawley regain weight by reduced energy expenditure without hyperphagia while the Long Evans have sustained hyperphagia without energy expenditure suppression [33].

Kaiyala *et al*. reported that *Lep*^*ob/ob*^ mice adjust energy expenditure but not food intake in response to changes in ambient temperature [34]. In contrast, wild type mice increase food intake when ambient temperature is decreased and reduce energy intake under thermoneutral conditions. In addition, wild type mice display a strong inverse relationship between ambient temperature and energy expenditure [34]. These data are consistent with our finding that *Lep*^*ob/ob*^ mice reduce energy expenditure but do not increase food intake in response to imposed weight reduction. *Lep*^*ob/ob*^ mice are known to be cold intolerant. Ideally these studies would have been carried out under thermoneutral conditions [35]. We speculate that the reductions in REE and TEE would still occur but that the mice would not save energy from the torpor contribution.

The CR *Lep*^*ob/ob*^ regained most of their lean mass within one week of release to *ad libitum* feeding, probably due to rehydrating of the muscle, whereas their fat mass took several weeks to reach the level of AL group. Mice regained lost lean mass quickly but did not exceed the amount of lean mass in the AL mice. Hambly *et al*. reported body composition of wild type mice post release from caloric restriction; like our *Lep*^*ob/ob*^, following release wild type mice regained lean mass more rapidly than the fat mass [7]. Both of these rapid recoveries in lean mass are likely due to both the rehydrating of muscle and the lower energy demand to gain 1 g of lean mass compared to gaining 1 g of adipose tissue.

*Lep*^*ob/ob*^ mice utilize energy in an age dependent but an apparent/composition mass (lean, fat or both) independent manner over the range of body mass/composition studied here. Unlike humans [4] or mice with intact leptin axis [6] Fig 8 indicates there is no apparent relationship between energy expenditure and fat, lean, or a combination of both in our *Lep*^*ob/ob*^ mice. Energy expenditure in *Lep*^*ob/ob*^ mice is only dependent on the feeding status of the mice; CR mice use significantly fewer calories than the AL controls but within either the CR or AL groups, in the size range that we observed, they use a non-detectably different number of calories regardless of size. Max Kleiber showed that across a wide range of body sizes, energy expenditure scales to the 3/4^th^ power of body mass [36]. In *Lep*^*obob*^ mice, over the size range studied, this relationship is not apparent due to methodological limitations. This is controversial. Using a multivariate regression model for 24h energy expenditure, Kaiyala *et al*. found that in leptin-deficient mice lean and fat mass were not significant contributors to variation in energy expenditure [12]. However, when leptin was administered to *Lep*^*ob/ob*^ mice, lean mass became an independent predictor of EE, suggesting that leptin is necessary to render the positive relationship between lean mass and EE detectable. Bolze *et al*, showed a positive correlation between body weight and REE in mice that lack leptin; they demonstrated this relationship within a small sample set but with very high sampling rate (readings every 9 minutes). It is possible that this high sampling rate uncovered a relationship that was not observed in the current study [14]. In the studies reported here, the lower energy expenditure in calorically restricted *Lep*^*ob/ob*^ mice is driven by a combination of torpor, a dramatic decrease in REE, and a decrease in NREE. In weight- reduced leptin-deficient mice the increased energy efficiency is more pronounced than in a weight reduced wild type mouse.

Insulin, in addition to its critical role in glucose homeostasis, is a known regulator of food intake and adiposity [37]. In leptin competent humans and mice, circulating insulin concentrations correlate directly with body weight and adiposity. However, in mice that lack leptin, this relationship is interrupted. [38–40]. Leptin administration suppresses beta cell insulin release, and reduces preproinsulin mRNA in multiple models including islets isolated from *Lep*^*ob/ob*^ mice [41, 42], rats [43] and humans [43, 44] and a rat pancreatic β-cell line [45]. Administration of exogenous leptin to *Lep*^*ob/ob*^ mice decreases circulating insulin and fasting plasma glucose concentrations even at doses that do not induce reductions in body weight [46]. In mouse models of lipodystrophy–characterized by severe hypoleptinemia and hyperinsulinemia–treatment with low doses of recombinant leptin significantly improved insulin sensitivity [47, 48] but chronic food restriction does not normalize circulating insulin concentrations [47, 48] suggesting that in lipodystrophic mice leptin modulates insulin sensitivity and glucose homeostasis independently of either food intake or body weight. In the current study, (and in leptin receptor-deficient fa/fa rats [49]), *ad libitum* fed *Lep*^*ob/ob*^ mice have elevated circulating insulin concentrations that are not normalized by caloric restriction. Hyperinsulinemia in AL *Lep*^*ob/ob*^ mice is not corrected by reducing fat mass (**Fig 4D**). The absence of circulating leptin in *Lep*^*ob/ob*^ mice is accompanied by a loss of the normal (**Fig 4E**) relationship between fat mass and circulating insulin.

In this study, *Lep*^*ob/ob*^ mice weight reduced by transient caloric restriction regained lost weight to the level of *ad libitum* controls. They did so by reduced energy expenditure; unlike wild type mice, there was no compensatory relative hyperphagia. Their reacquisition of lost body fat suggests that factors other than leptin are involved in regulating body weight of the *Lep*^*ob/ob*^ mouse. The adiposity “set point” in the *Lep*^*ob/ob*^ mouse is apparently higher than that of a wild type mouse, and this higher level is defended when the mouse is challenged with caloric restriction. Other circulating and/or neural factors inform the CNS about aspects of energy intake and somatic energy stores. Some of these have their predominant effects on either energy intake (insulin, ghrelin, CCK, GLP-1, PYY) or expenditure (thyroid hormone). Others almost certainly exist [50]. For example, when a wild type mouse is parabiosed to a leptin receptor deficient mouse (*Lep*^*db/db*^) with high (but adipose mass-appropriate) circulating concentrations of leptin the wild type mouse starves to death [51, 52]. However, administering a similar amount of leptin to a wild type mouse only transiently reduces food intake and the animal does not starve [53] [53]. These results support the possible existence of another circulating factor–a starvation signal–that is present in a *Lep*^*db/db*^ because the mouse is unable to properly sense its energy stores. Such a signal might account for the hypometabolic response of the *Lep*^*ob/ob*^ mouse to weight reduction.

## Supporting information

S1 Fig Supplemental file on EE. Explanation of why the EE post release was calculated from food intake and change in body composition instead of just output from the TSE. EE from indirect calorimeter after *ad libitum* release. Energy expenditure during calorie restriction was measured in the TSE metabolic chambers estimated using the Weir equation. Included are TEE–total energy expenditure and REE–resting energy expenditure.
(DOCX)

S1 File This file has 6 source data files included in manuscript. The six included files are: 1. 050515 OB Calo Group 1 during CR Deleted. Raw file from the TSE machine from the first group of mice in calorimeter while on caloric restriction, food intake was deleted as the food was weighed and placed in the cages twice daily without using the TSE scale. 2. 051415 OB Calo Group 1 after CR release. Raw file from the TSE machine from the first group of mice in calorimeter after the caloric restriction was lifted and mice were allowed to eat *ad libitum*. 3. 052715 OB Calo Group 2 after CR release. Raw file from the TSE machine from the second group of mice in calorimeter after the caloric restriction was lifted and mice were allowed to eat *ad libitum*. 4. Insulin Elisa and Glucose. Insulin and Glucose raw data including standard curves used to calculate the values used in the manuscript. 5. OB study mice 022116. Raw data from food intake, body composition, body temperature, and body weight. and 6. TEE and Tot Mvt ob by mouse during and post CR. Raw data that shows how we ordered and averaged data to generate Fig 6C and 6D.
(ZIP)
