# Supplementary material for: Energy homeostasis in leptin deficient Lepob/ob mice
Source: PLoS One. 2017 Dec 20;12(12):e0189784. doi: 10.1371/journal.pone.0189784 (PMC5738099; doi:10.1371/journal.pone.0189784)
Supplement: S1 Fig — Explanation of why the EE post release was calculated from food intake and change in body composition instead of just output from the TSE. EE from indirect calorimeter after ad libitum release. Energy expenditure during calorie restriction was measured in the TSE metabolic chambers estimated using the Weir equation. Included are TEE–total energy expenditure and REE–resting energy expenditure. (DOCX) [file pone.0189784.s001.docx]

**Supplemental Data on EE from calorimeter after CR was lifted**

After CR was removed and mice were allowed to eat *ad libitum*, the CR mice rapidly gained weight despite the lack of hyperphagia compared to the *ad libitum* mice. Since the mice are rapidly gaining weight, the post release RQ is significantly above 1.0 (figure 7B). The Weir equation is valid from an RQ between 0.7 and 1.0, and relies on the fact that burning carbohydrate produces an RQ of 1.0 and pure fat an RQ of 0.71. An RQ of 1.0 the Weir equation generates 5.05 kcals/l of O_2_ consumed; at 0.71, the formula generates 4.69 kcals/l O_2_ consumed. At values above 1.0, it overestimates the kcals consumed; the food that is being utilized to generate this energy still has a DQ of 0.89, the excess RQ comes from lipogenesis. Values above 1.0 rarely practically occur (have been seen in animals about to enter hibernation). Since the post-CR release mice have RQs above 1.0, the equation is slightly overestimating the EE in these mice and we think that, in this rare circumstance, the energy balance method is more appropriate. After release from CR, by calorimetry, there is a slight, but not significant, decrease in TEE and REE. The TSE system collected data every 17 minutes.
